# Management of juvenile and aneurysmal bone cysts: a systematic literature review with meta-analysis

**DOI:** 10.1007/s00068-022-02077-9

**Published:** 2022-08-21

**Authors:** Jonas A. Strohm, Peter C. Strohm, Jan Kühle, Hagen Schmal, Jörn Zwingmann

**Affiliations:** 1 Albert-Ludwigs-University of Freiburg, Freiburg, Germany; 2 Clinic for Orthopedics and Trauma Surgery, Klinikum Bamberg, Bamberg, Germany; 3 Department of Orthopedic and Trauma Surgery, University of Freiburg Medical Center, Freiburg, Germany; 4 Clinic of Orthopedic and Trauma Surgery, Oberschwabenklinik Ravensburg, Ravensburg, Germany

**Keywords:** Bone cyst, Aneurysmal, Juvenile, Meta-analysis

## Abstract

**Purpose:**

Numerous approaches to the management of juvenile and aneurysmal bone cysts (ABC) are described in the specialist literature together with discussion of the associated healing and recurrence rates. Since there is currently no evidence-based treatment standard for these conditions, the aim of this systematic literature review with meta-analysis was to examine the different management approaches, evaluate the corresponding clinical outcomes and, as appropriate, to formulate a valid treatment recommendation.

**Methods:**

A systematic search on OVID Medline^®^ based on a pre-existing search strategy returned 1333 publications. Having defined inclusion and exclusion criteria and analysis of the relevant full texts, 167 publications were included in the descriptive analysis and 163 in the meta-analysis. For this purpose, different subgroups were created, based on the type of cyst and the therapeutic procedure. Those subgroups were then analysed in relation to their healing rates, the number of recurrences and complication rates.

**Results:**

For aneurysmal bone cysts, both surgical removal and Doxycycline injection lead to excellent outcomes (98% healing) and low recurrence rates (6% and 11% resp.). Curettage (91% healing), including its combination with autologous cancellous bone graft (96% healing), showed very good healing rates but higher recurrence rates (22% and 15%, resp.), which were however improved by preoperative selective arterial embolization. A critical view must be taken of radiotherapy (90% healing) and the injection of alcohol (92% healing) because of their high complication rates (0.43/cyst and 0.42/cyst, resp.). In the management of juvenile bone cysts, surgical interventions like curettage and cancellous bone graft (87% healing) are far superior to non-surgical approaches (51% healing), furthermore, the application of autologous cancellous bone graft reduced the recurrence rate (3% recurrence) compared to curettage alone (20% recurrence). In subgroup analysis, treatment by ESIN was found to produce excellent outcomes (100% healing), though the patient collectives were small.

**Conclusion:**

Surgical procedures to treat aneurysmal bone cysts appear to be the method of choice whereby Doxycycline injection may be an alternative. A surgical approach should be preferred in the treatment of juvenile bone cysts.

## Introduction

Juvenile and aneurysmal bone cysts are classed as benign, tumour-like bone lesions [1, 2] and are among the most common manifestations in childhood and adolescence.

Juvenile bone cysts (synonym: simple, unicameral bone cyst) usually build a cavity in the bone, covered with a thin endothelial layer/film and filled with clear fluid. These cysts occur most frequently at the proximal metaphysis of the humerus, femur, tibia and calcaneus [3]. They may often remain asymptomatic for a long time and, in almost two-thirds of cases, they are only discovered in the context of pathological fracture [4, 5].

Aneurysmal bone cysts are characterized by the formation of multiple cavities, filled with blood, proliferating fibroblasts and giant cells and septated by bone trabeculae and fibrous connective tissue septa [6, 7]. Almost half of them are meta-diaphyseal and the remainder predominantly metaphyseal occurring in the long bones (primarily in the lower extremities) [6, 7]. Other frequent locations are the spine and pelvis [8–11]. They generally become clinically apparent due to non-specific general symptoms like pain, swelling or restricted movement in the adjacent joint [11].

Unlike in the past when the only treatment of juvenile bone cysts was almost exclusively by surgical intervention, reports in the specialist literature of the past 40 years have increasingly described different, sometimes complex, treatment procedures that vary greatly in their level of invasiveness.

Surgical, sometimes multimodal, treatment approaches may be comprised of curettage and postoperative defect filling with cancellous bone or cement, insertion of an elastic stable intramedullary nail (ESIN) or the use of chemical adjuvants like phenol, for example [5, 12–14]. Injection of alcohol or steroids, percutaneous or intraoperative radiotherapy or the (super-)selective arterial embolization are examples of less invasive procedures [15–17]. There are increasing references in the literature to treatments that involve filling the defect with allogenic or xenogenic materials and treatments with newer substances like the (off-label) use of Denosumab, a monoclonal antibody [18, 19].

Currently, no evidence-based treatment standard is offered by the literature, therefore, the aim of this review was to compare different treatment approaches by subgroup analysis of healing, recurrence and complication rates based on a systematic literature search with subsequent meta-analysis. In addition, we discuss aspects of clinical feasibility in order to offer, if possible, a valid treatment recommendation.

## Methods

### Literature research and search strategy

**Fig. 1:**
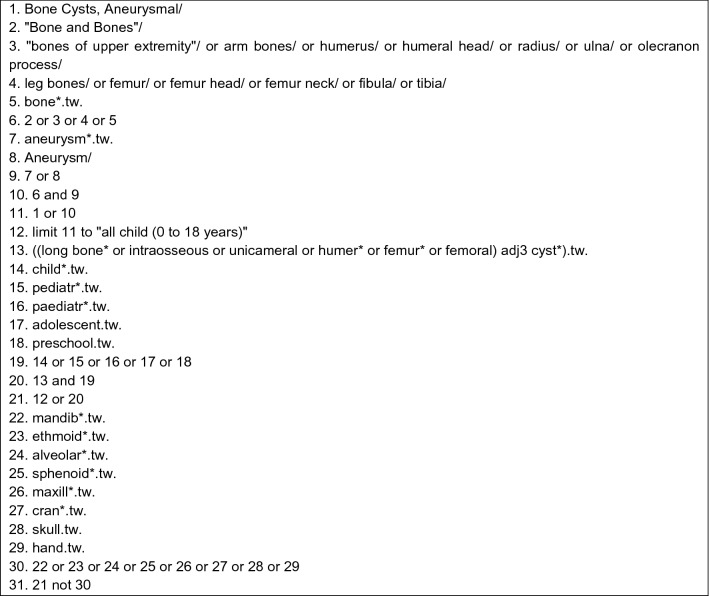
Search strategy through Ovid SP

Data collection for the systematic literature search was acquired through the Ovid SP platform, which accesses the Medline database. Our search strategy was conducted as shown in Fig. 1 and resulted in *n* = 1333 publications.

### Study selection

The abstracts of the 1333 publications were reviewed for their suitability according to our own specifically defined inclusion and exclusion criteria. All prospective and retrospective clinical follow-up studies and study subgroups reporting on the management and treatment outcomes of aneurysmal and juvenile bone cysts were included (inclusion criteria). Conversely, all overviews, review articles, case reports, biomechanical and radiological studies as well as all articles that were not written in English or German were excluded. The follow-up period was set at > 12 months (exclusion criteria).

In a next step, the full texts of the articles thus identified (*n* = 186) were evaluated and relevant data was entered into an Excel table. A further 15 publications that had not been found through literature search on the Ovid platform were added as they were highly relevant to this study.

**Fig. 2:**
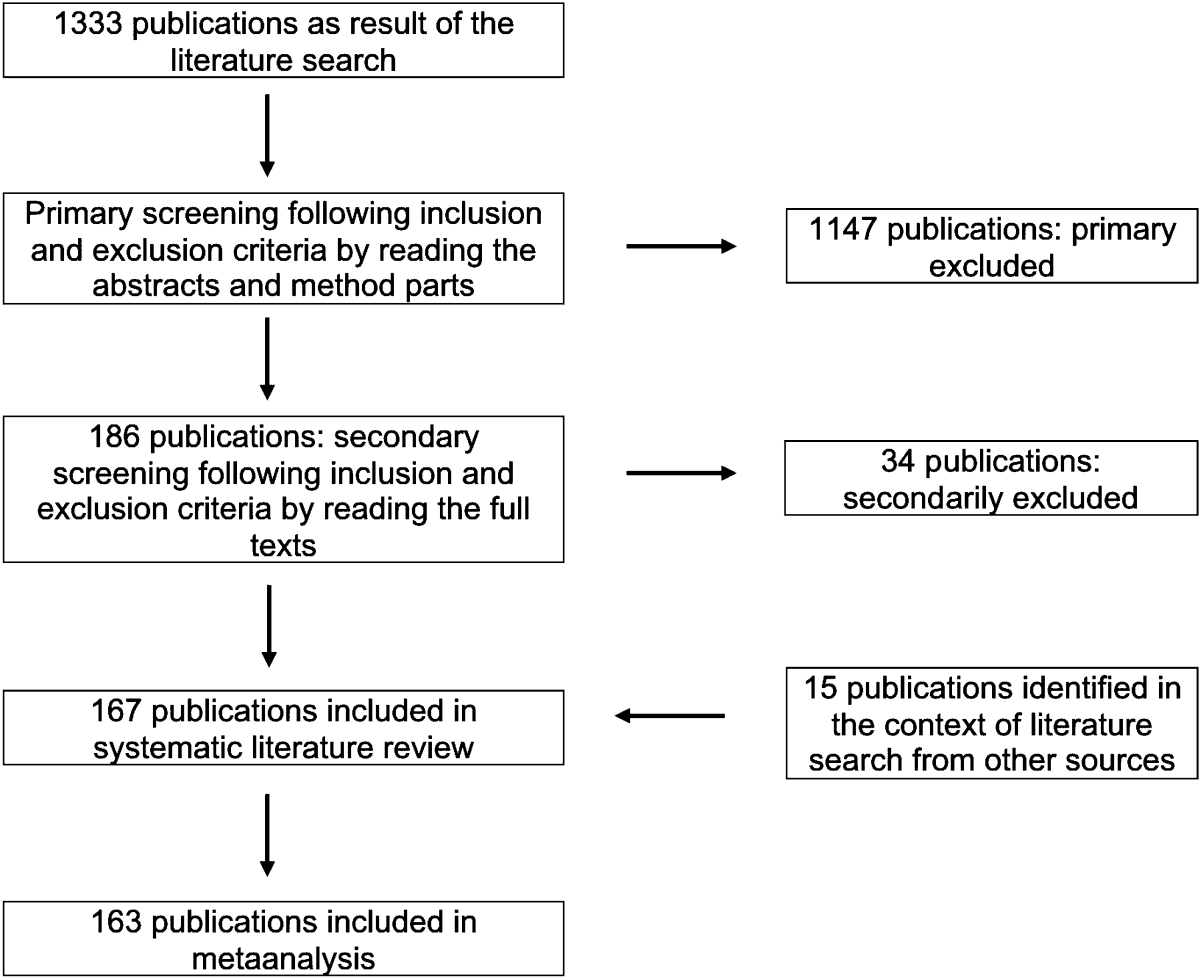
PRISMA Chart

The publication selection process is shown in Fig. 2 in accordance with the 2009 PRISMA Statement [20].

### Data acquisition

The data extracted (if reported) from the 167 publications in the study as listed below was entered in an Excel table:

1. Name of the author(s), year of publication, name of the journal
2. Study size/study type/level of evidence
3. Number/type of cysts
4. Number of pathological fractures
5. Therapeutic interventions
6. Epidemiological codes (age, gender)
7. Follow-up in months
8. Number of recurrences/treatment of recurrent cysts
9. Healing (total healing; complete/partial healing)
10. Localization of cysts
11. Stage score before and after treatment (acc. Enneking/Capanna)
12. Number of refractures
13. Number of complications

Based on the classification published in the Journal of Bone and Joint Surgery in 2003, the studies were assessed for their Level of Evidence, if it were not already cited in the article [21]. The analysis revealed one study with an evidence score of 2, 27 studies with a score of 3, and 139 studies with a score of 4.

There were 3 prospective studies, 163 retrospective studies and one study with a mixed retro- and prospective study design.

### Evaluation

In addition to tabular presentation of data with evaluation of the descriptive numerical entries using Microsoft Excel for Mac (Version 16.44), a subsequent meta-analysis was performed with the Open Source package “R” (Version 4.0.4) in R Studio (Version 1.2.1106) [22].

This facilitated subgroup analysis of healing, recurrences and complications for each type of cyst for each treatment method as well as creation of the corresponding forest plots.

The “R” package meta with the function metaprop was employed for the meta-analysis of proportions, including subgroup analysis for recurrences and healing [23]. Subgroup analysis for complications was visualized by meta-analysis of rates using the metarate function.

Since numerous different treatment procedures were described, frequently with only a small collective of cysts, we decided that we would only report and discuss the outcomes of procedures reported in at least three studies in which at least 30 cysts were treated. Due to its increasing relevance, we made an exception for treatment of aneurysmal bone cysts with Denosumab and treatment of juvenile bone cysts with ESIN.

Studies that offered no clear definition of’ ‘healing’ of the documented cysts were only included in the analysis of recurrences.

## Results

A total of 118 publications, dedicated exclusively to the treatment of aneurysmal bone cysts, and 45 publications, dedicated only to the treatment of juvenile bone cysts were included in the descriptive analysis and the meta-analysis (*n* = 163). A further 4 publications concerned with the treatment of both types of bone cyst were included in the descriptive analysis only.

Overall, a collective was achieved consisting of 3467 aneurysmal bone cysts, 2227 juvenile bone cysts and 133 that could not be definitively assigned to one group or the other.

### Results of the descriptive analysis

For all the different treatments of all juvenile bone cysts (*n* = 2227) a total of 69.1% (n = 1535) healed completely. Of the aneurysmal bone cysts (*n =* 3467) only 52.2% (*n =* 1809) went on to complete healing. 9.5% (*n =* 212) of the juvenile cysts and 15.1% (*n =* 523) of the aneurysmal cysts recurred. Although 40.9% (*n =* 911) of the juvenile cysts caused a pathological fracture and the refracture rate during the course of treatment was 4.1% (*n =* 92), pathological fracture only occurred in 5.2% (*n =* 180) of the aneurysmal cysts with refracture at only 1% (*n =* 34).

### Results of the meta-analysis

**Fig. 3:**
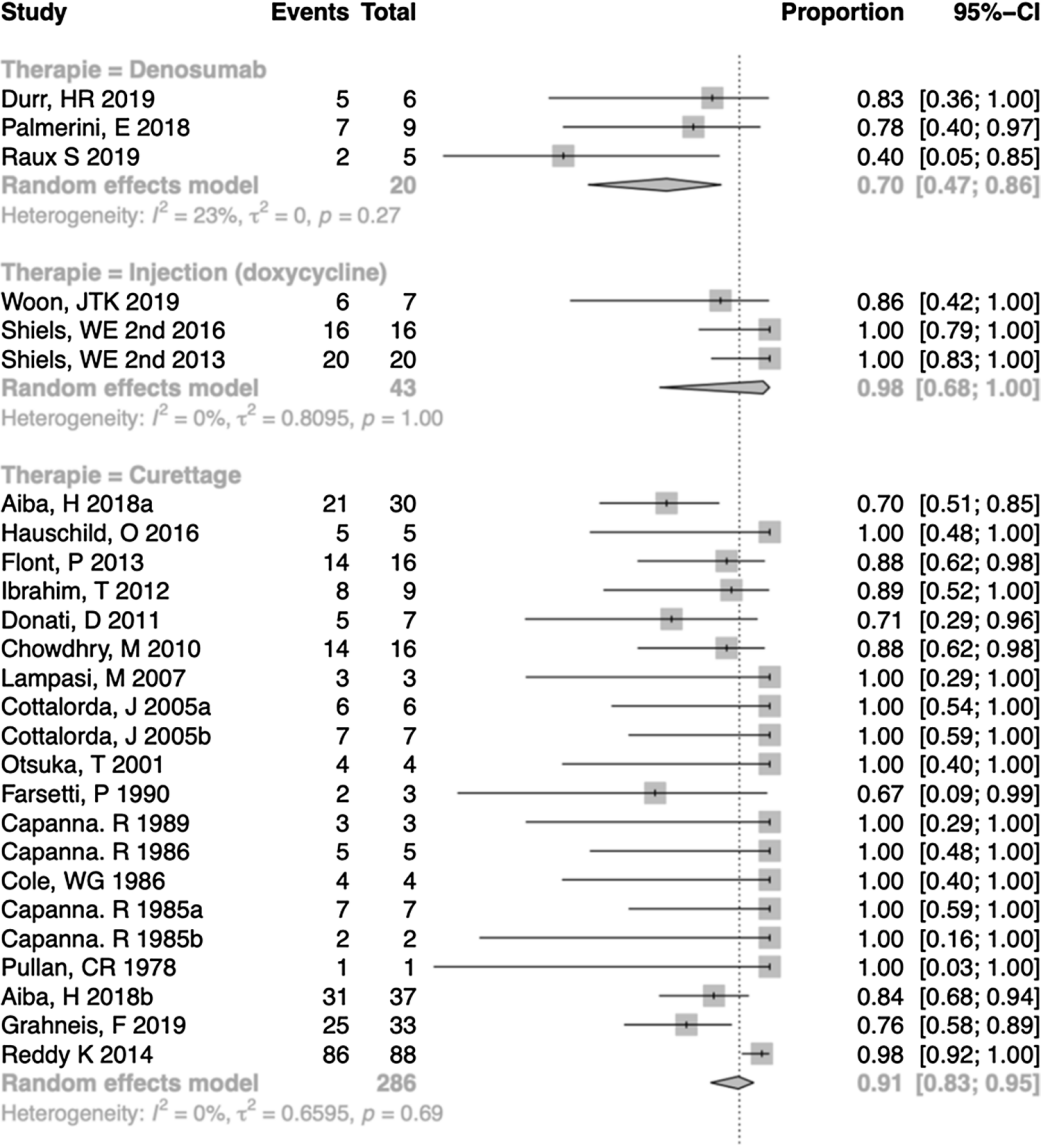
Section of forest plot for healing of aneurysmal bone cysts

**Fig. 4:**
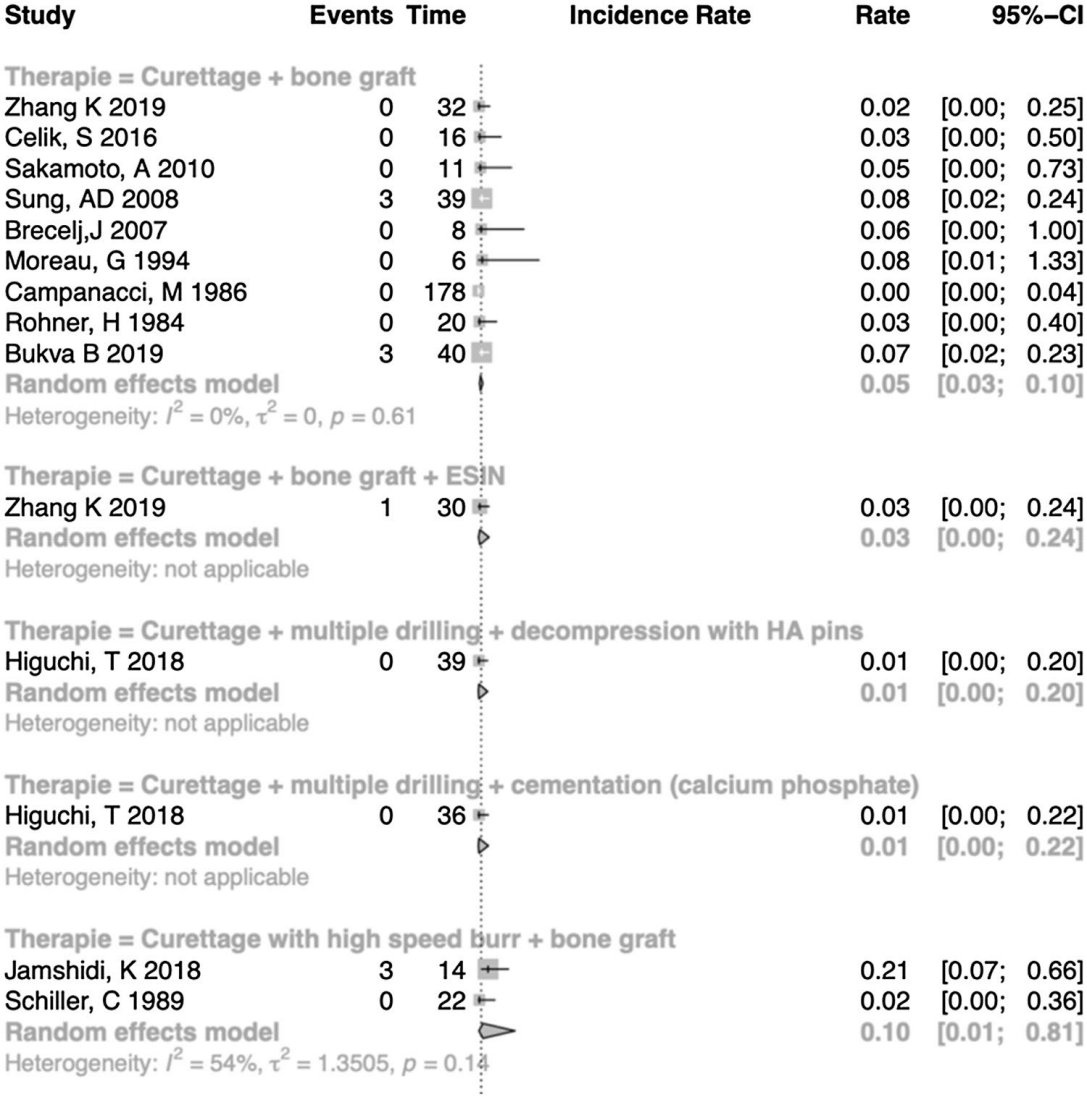
Section of forest plot for complication rates of juvenile bone cysts

To simplify visualization and because of the numerous subgroups in our meta-analysis with correspondingly large forest plots, we decided to set out the results in tables. As an example, Fig. 3 shows a section of the forest plot on healing of aneurysmal bone cysts. Figure 4 shows a section of the forest plot for complications in the treatment of juvenile bone cysts.

#### Results for healing of juvenile bone cysts

As is clear from Table 1, both curettage with subsequent cancellous bone grafting (87% healing of 330 cysts) and steroid injections (78% healing of 786 cysts) are procedures that achieve good outcomes, whereby, for the latter, the relevant studies showed substantial heterogeneity at a statistically significant *p* value (*p* < 0.01). Non-surgical management of cysts only led to healing in half of all cases, whereby ‘non-surgical ‘ here refers to a ‘watch-and-wait ‘ approach.

**Table 1:** Results of meta-analysis of juvenile bone cyst healing after different treatments

Therapy	Studies (*n*)	Cysts (*n*)	Effect-estimate and 95% CI	*I*^2^ in %	*p*
Curettage + bone graft	8	330	0.87 [0,59; 0,97]	58	0.02
Conservative	4	115	0.51 [0,29; 0,72]	60	0.06
Injection Steroids	16	786	0.78 [0,60; 0,90]	92	< 0.01
ESIN	2	65	1.00 [0,00; 1,00]	0	1.,00
Injection bone marrow + DBM	2	65	0.75 [0,64; 0,84]	21	0.26

**Table 2:** Results of meta-analysis of the number of recurrences of juvenile bone cysts after different treatments

Therapy	Studies (*n*)	Cysts (*n*)	Effect-estimate and 95% CI	*I*^2^ in %	*p*
Curettage + bone graft	9	350	0.03 [0,00; 0,27]	0	0.97
Conservative	5	116	0.02 [0,00; 0,84]	0	1.00
Injection Steroids	16	786	0.00 [0,00; 0,05]	0	0.99
Curettage	4	65	0.20 [0,12; 0,31]	0	0.75
Surgery + bone graft	3	73	0.07 [0,03; 0,15]	0	0.86

#### Results for recurrence of juvenile bone cysts

Apart from the excellent outcomes achieved by (repeated) steroid injections (0% recurrence of 786 cysts), very good results were also observed for curettage combined with subsequent cancellous bone graft (3% recurrence of 350 cysts). Even undetermined surgical interventions combined with cancellous bone graft very good outcomes were recorded (7% recurrence of 73 cysts). If healing was achieved by non-surgical treatment, the number of recurrences was also very low (2% recurrence of 116 cysts). Curettage alone was accompanied by the highest number of recurrences (20% recurrence of 65 cysts) (Table 2).

#### Results for juvenile bone cyst complications

The findings showed that complication rates for all the different treatment procedures for juvenile cysts were overall very low (see Table 3). Surgical and non-surgical interventions alike led to very few complications: curettage with cancellous bone graft (0.05 complications/cyst for 350 cysts), ESIN (0.02 complications/cyst for 65 cysts), non-surgical treatments (0.05 complications/cyst for 116 cysts). Steroid injections were also associated with a very low complication rate (0.07 complications/cysts for 786 cysts), whereby there was also substantial heterogeneity at a statistically significant *p* value (*p* < 0.01).

**Table 3:** Results of meta-analysis of complication rates for juvenile bone cysts after different treatments

Therapy	Studies (*n*)	Cysts (*n*)	Effect-estimate and 95% CI	*I*^2^ in %	*p*
Curettage + bone graft	9	350	0.05 [0,03; 0,10]	0	0.61
Conservative	5	116	0.05 [0,01; 0,24]	31	0.22
Injection Steroids	16	786	0.07 [0,04; 0,13]	66	< 0.01
Curettage	4	65	0.05 [0,01; 0,19]	0	0.66
ESIN	2	65	0.02 [0,00; 0,12]	0	0.63
Surgery + bone graft	3	73	0.02 [0,00; 0,11]	0	0.82
Injection bone marrow + DBM	2	65	0.03 [0,01; 0,14]	0	0.48

#### Results for healing of aneurysmal bone cysts

The data in Table 4 clearly show that the two injection procedures, i.e. with Doxycycline (98% healing of 43 cysts) or alcohol (92% healing of 299 cysts), and cyst resection (98% healing of 94 cysts) yielded very good to excellent outcomes. Surgical intervention also demonstrated very good outcomes, except for curettage with cancellous bone graft (80% healing of 229 cysts). In this case, it must be considered that, based on a statistically significant *p* value (*p* < 0.01), there was substantial heterogeneity of the individual studies. Although radiotherapy alone (90% healing of 52 cysts) and selective arterial embolization (81% healing of 211 cysts) did deliver good outcomes, treatment with Denosumab (70% healing of 20 cysts) led to the poorest results in terms of healing in this meta-analysis.

**Table 4:** Results of meta-analysis of aneurysmal bone cyst healing after different treatments

Therapy	Studies (*n*)	Cysts (*n*)	Effect-estimate and 95% CI	*I*^2^ in %	*p*
Denosumab	3	20	0.70 [0,47; 0,86]	23	0.27
Injection Doxycycline	3	43	0.98 [0,68; 1,00]	0	1.00
Curettage	20	286	0.91 [0,83; 0,95]	0	0.69
Selective arterial embolization	15	211	0.81 [0,75; 0,86]	0	0.86
Surgery	6	64	0.95 [0,63; 1,00]	0	0.99
Radiotherapy	10	52	0.90 [0,67; 0,97]	0	1.00
Curettage + autograft	5	57	0.96 [0,53; 1,00]	41	0.15
Curettage + bone graft	10	229	0.80 [0,65; 0,89]	61	< 0.01
Injection Alcohol	13	299	0.92 [0,84; 0,97]	25	0.19
Resection	13	94	0.98 [0,79; 1,00]	0	1.00
Resection + bone graft	4	31	0.94 [0,78; 0,98]	0	0.96

#### Results for the recurrence of aneurysmal bone cysts

As shown in Table 5, the number of recurrences after Doxycycline injection (6% recurrence of 43 cysts) and after alcohol injection (5% recurrence of 302 cysts) was very low. Very good outcomes were achieved by curettage combined with selective arterial embolization (10% recurrence of 52 cysts) and cyst resection (11% recurrence of 246 cysts).

**Table 5:** Results of meta-analysis of the number of recurrences of aneurysmal bone cysts after different treatments

Therapy	Studies (*n*)	Cysts (*n*)	Effect-estimate and 95% CI	*I*^2^ in %	*p*
Denosumab	4	29	0.22 [0,08; 0,49]	29	0.24
Injection Doxycycline	3	43	0.06 [0,02; 0,18]	0	0.98
Curettage	33	522	0.22 [0,18; 0,26]	0	0.46
Curettage + allograft	5	136	0.23 [0,16; 0,30]	0	0.84
Curettage + autograft	8	87	0.15 [0,07; 0,30]	27	0.22
Resection	27	246	0.11 [0,07; 0,16]	0	0.89
Selective arterial embolization	16	228	0.16 [0,10; 0,25]	26	0.17
Surgery	9	87	0.15 [0,09; 0,25]	0	0.87
Radiotherapy	14	56	0.23 [0,13; 0,37]	0	0.85
Curettage + bone graft	22	468	0.29 [0,21; 0,39]	63	< 0.01
Injection Alcohol	14	302	0.05 [0,03; 0,10]	10	0.35
SAE + Curettage	4	52	0.10 [0,04; 0,23]	0	0.54
Surgery + bone graft	4	59	0.17 [0,09; 0,30]	0	0.65
Resection + bone graft	6	53	0.21 [0,09; 0,41]	30	0.21
Curettage + radiotherapy	7	47	0.17 [0,09; 0,32]	0	0.85

The treatment of curettage combined with autogenic cancellous bone graft led to a relatively low recurrence rate (15% recurrence of 87 cysts). In contrast, a distinctly higher number of recurrences were found for curettage alone (22% recurrence of 522 cysts), curettage with allogenic cancellous bone graft (23% recurrence of 136 cysts), and curettage with (undefined) cancellous bone graft (29% recurrence of 468 cysts), whereby, for the latter, there was substantial heterogeneity based on a statistically significant *p* value (*p* < 0.01).

In this meta-analysis, radiotherapy was also associated with a fairly high number of recurrences (23% recurrence of 56 cysts).

#### Results for aneurysmal bone cyst complications

Low complication rates were found for curettage alone (0.09/cyst for 522 cysts) and combinations of curettage with cancellous bone graft or selective arterial embolization (0.09/cyst for 52 cysts) (see Table 6).

**Table 6:** Results of meta-analysis of complication rates for aneurysmal bone cysts after different treatments

Therapy	Studies (*n*)	Cysts (*n*)	Effect-estimate and 95% CI	*I*^2^ in %	*p*
Denosumab	4	29	0.27 [0,13; 0,56]	0	0.74
Injection Doxycycline	3	43	0.08 [0,02; 0,24]	0	0.98
Curettage	33	522	0.09 [0,06; 0,14]	0	0.78
Curettage + allograft	5	136	0.11 [0,03; 0,39]	45	0.12
Curettage + autograft	8	87	0.08 [0,03; 0,20]	0	0.75
Resection	27	246	0.13 [0,08; 0,20]	0	0.88
Selective arterial embolization	16	228	0.12 [0,07; 0,18]	0	0.50
Surgery	9	87	0.16 [0,09; 0,28]	0	0.97
Radiotherapy	15	68	0.43 [0,29; 0,64]	0	0.96
Curettage + bone graft	22	468	0.07 [0,04; 0,11]	0	0.71
Injection Alcohol	14	302	0.42 [0,28; 0,64]	79	< 0.01
SAE + Curettage	4	52	0.09 [0,02; 0,38]	1	0.39
Surgery + bone graft	4	59	0.12 [0,04; 0,34]	2	0.38
Resection + bone graft	6	53	0.21 [0,09; 0,46]	13	0.33
Curettage + radiotherapy	7	47	0.33 [0,17; 0,66]	7	0.37

The injection of alcohol (0.42/cyst for 302 cysts) had a distinctly higher complication rate compared to injection of Doxycycline (0.08/cyst for 43 cysts) as did radiotherapy (0.43/cyst for 68 cysts). For alcohol injection there was also a statistically significant *p* value (*p* < 0.01), whereby the studies were fairly heterogeneous.

## Discussion

The aim of this study was to investigate the high number of different procedures described in the literature for the treatment of aneurysmal and juvenile bone cysts and to apply subgroup analysis to identify their clinical value with an emphasis on healing, recurrence and complication rates. It is difficult to arrive at a sound recommendation based on our analysis due to the substantial variations in the treatment approaches that are reflected in the enormous number of subgroups often having very few patients or small study collectives, not to mention the potential of an associated publication bias. In addition, individual studies describing one or other of the procedures were extremely heterogeneous. Nevertheless, it is possible to make a valid statement about some of the treatment approaches based on the number of studies and their corresponding patient collectives.

### Therapeutic treatment of juvenile bone cysts

#### Non-surgical methods to treat juvenile bone cysts

Non-surgical management of juvenile bone cysts yielded the poorest results with only 51% healed cysts identified by our meta-analysis. It appeared that a number of factors had an important influence on successful healing such as prior pathological cyst fracture and/or prior biopsy as well as the size and extent of the cyst [24].

Non-surgical management bears increased risks of (re)-fracture, a long period of reduced physical activity and possible splinting of the extremity. Especially If the cyst occurs at the proximal femur, surgical management is strongly recommended to avoid the risk of femoral head necrosis.

Arguments in favour of non-surgical treatment of juvenile bone cysts include very low complication rates and very few recurrences as confirmed by our meta-analysis, whereby 95% CI from 0 to 84% should be considered critically. In this regard, recurrence rates of 18–19% are cited in the literature whereby the figures are distinctly higher for female patients in the first decade of life [25]. It would seem essential to give each case careful consideration taking into account the patient’s previous history and assessing the patient’s desire to have surgery.

#### Surgical management of juvenile bone cysts

Curettage of juvenile cysts as an isolated procedure achieved satisfactory outcomes (79% healing) within a small cyst collective of 14 cysts. The application of cancellous bone graft after curettage substantial increased the success rate (87% healing). Although, the individual studies varied greatly, findings from our other subgroup analyses of curettage combined with different bone substitutes also revealed good to very good outcomes. Furthermore, the high recurrence rates for curettage alone (20% recurrence) were significantly decreased when it was combined with cancellous bone graft (3% recurrence). Sung et al. reported on 39 patients in whom curettage alone resulted in a failure rate of 64%. They identified very young age frequently coinciding with aggressive, fast growing cysts and femoral location as prognostically unfavourable for healing [26].

In addition to the factors named above, it is important to mention the skill and technical know-how of the surgeon, since these parameters can greatly improve the chances of a good outcome [27].

In this meta-analysis, there was a paucity of studies on which to base an evaluation of ESIN treatment whether as an isolated method or in combination with curettage and subsequent defect filling or in the context of multimodal therapeutic concepts. However individual subgroup analysis showed a 100% healing rate. Certain factors are assumed to be conducive to the progress of healing such as immediate stability for exercise and loading, low postoperative fracture risk and continuous decompression of the bone cyst by intramedullary nailing [28].

#### Injection methods to treat juvenile bone cysts

Steroid injections showed a moderate success rate (78% healing) in our meta-analysis. There was also considerable heterogeneity (*p* < 0.01) across the individual studies, which means that the outcomes of each study differed greatly in some instances. This might be attributed to wide variation in the reported endpoints of treatment after steroid injection and the lack of a standard follow up time. Interestingly, in a study by Di Bella et al., the healing rate was 21% for 143 patients for a single injection, but this increased to 38% for three consecutive injections [29]. Other authors were able to attain a healing rate of 58.6% after 24 months by increasing the number of steroid injections [5]. Capanna et al. propose an average of 3–4 steroid injections over a period of at least 12 months before osseous consolidation will be seen at the cyst site [12, 30].

Given this variation and the heterogeneity of the outcomes as well as the lack of consistent endpoints in terms of treatment duration and number of injections, it is challenging to draw any clear conclusions. Nonetheless, our findings derived from the meta-analysis indicate that surgical intervention will be more appropriate than steroid injection, especially against the backdrop of a repeated need for general anaesthesia and risk of fracture due to insufficient loading stability during the course of healing [31].

### Management procedures for aneurysmatic bone cysts

#### Surgical procedures for aneurysmal bone cysts

Simple curettage yielded good results (91% healing) as well as low complication rates (9%) but a relatively high number of recurrences (22% recurrence) in our meta-analysis. Similar outcomes were echoed in the literature, e.g. Tilman et al. and Ruiter et al. who reported recurrence rates of 28–34% [32, 33]. Here, critical differentiation is crucial with regard to technique and skill in curettage. Endoscopic curettage or the use of a high-speed burr, for example, produced much lower recurrence rates of 10% in the treatment of large patient collectives [7, 34]. At the same time, minimally invasive procedures reduce the number of complications.

Curettage in the context of a multimodal management approach, e.g. in combination with cryotherapy and subsequent cancellous bone graft or ESIN with application of synthetic bone substitute were found within their subgroups to yield excellent outcomes and much lower recurrence rates (4–5% recurrence) [35, 36]. However, too few studies were available to permit the formulation of a valid recommendation.

The situation was similar for curettage with subsequent defect filling with autologous cancellous bone. This method also showed better outcomes (96% healing) and lower recurrence rates (15% recurrence) compared with simple curettage but more studies are needed to allow any precise recommendation.

Resection/excision of cysts produced excellent outcomes (98% healing) and few recurrences (11% recurrence). Unfortunately, the term ‘resection’ is not used consistently so it often refers to interventions of varying extent that may be more or less drastic. In this regard, Harms et al. stated that radical cyst resection might lead to limb shortening and axial deviation, therefore, it should be evaluated critically before proceeding in young patients with an open growth plate [37].

In our meta-analysis the term ‘surgery’ refers to surgical interventions of varying complexity that were frequently reported in the included studies and usually applied in the management of spinal bone cysts. Here, good outcomes could be confirmed as for all other surgical interventions.

#### Injection methods in the treatment of aneurysmal bone cysts

Although injection methods with alcohol (92% healing, 5% recurrence) and with Doxycycline (98% healing, 6% recurrence) both led to very good outcomes, they must be considered critically for the following reasons. Firstly, in our meta-analysis alcohol injections were the worst affected by complications (0.42/cysts), predominantly local indurations and hypopigmentation of the skin around the puncture site [38, 39]. Secondly, as already mentioned under injection methods for juvenile cysts, a median of 2–4 treatments are required before healing is achieved. Brosjö et al. reported up to 11 injections for their patients, each of which was associated with the renewed risk of general anaesthesia [40]. Cruz et al. described in their meta-analysis on percutaneous treatments of primary aneurysmal bone cyst good results of healing and low recurrence rates, similar to the results of our meta-analysis [41]. As we described above, based on our findings, the complications rates of these methods are relatively high. In their study, they found an overall complication rate of about 17%. In our meta-analysis we found complication rates of 0.08/cyst for the injection of Doxycycline, 0,12/cyst for the selective arterial embolization and the highest complication rate of 0,42/cyst for the injection of alcohol. These findings and the necessity of repeated injections with the risk of general anaesthesia are the reason why we do not recommend this therapy, in contrast to Cruz et al..

Although evaluation of the subgroups did reveal that other injection procedures (calcitonin, steroids) resulted in good outcomes, the question arises of whether consolidation of the cysts might already be achieved by decompression alone and that the injected substance is not crucial to success. Reddy et al. treated 102 patients by so-called “curopsy” , i.e. biopsy with a curative intention. After a median follow up of 14 months 81% of cysts had healed [34].

#### Other procedures for the treatment of aneurysmal bone cysts

Radiotherapy alone does deliver very good outcomes (90% healing), it is ,however, associated with a high risk of recurrence (23% recurrence), high complication rate (0.43/cysts) and requires a treatment regimen that lasts for weeks or months [17]. Possibly, radiotherapy could be employed as an adjuvant procedure to other therapeutic interventions or be considered in cases of recurrence after surgery. In these situations, it is important to be acutely aware of the risk of malignancy associated with repeated exposure to radiation and to avoid this whenever possible [17, 42]. This is of key importance in the case of bone cysts in children.

Although selective arterial embolization in isolation led to poorer outcomes (81% healing) compared to other methods described here, it has been described in 3 large studies by Terzi et al. and Rossi et al. as a suitable procedure for the management of aneurysmal bone cysts of the spine. An essential requirement is however the adequate experience and technical know-how of the surgeon [16, 43, 44]. As mentioned above in the context of radiotherapy and the various injection procedures, selective arterial embolization also requires numerous, frequent treatment sessions involving repeated exposure to radiation in order to achieve healing [45].

A review of smaller studies in our subgroups indicated that selective arterial embolization with subsequent curettage and/or resection with or without cancellous bone graft, produced good outcomes and, above all, lower recurrence rates compared to surgical intervention alone. Nevertheless, the number of studies is insufficient to permit any conclusive statement. A neo-adjuvant application of selective arterial embolization prior to a planned operation does seem to significantly reduce the quantity of blood lost intraoperatively [46].

It is not possible to make a clear statement on the use of the monoclonal antibody Denosumab even though it is increasingly appearing in up-to-date literature because the number of cases appearing in the included studies of our meta-analysis was very limited. Findings so far have not indicated convincing outcomes (70% healing) but do show a tendency towards a higher recurrence rate (22% recurrence) for intravenous or subcutaneous application alone. Potential for this method has been identified in the context of (neo)-adjuvant administration as part of surgical intervention. This approach aims to alleviate symptoms and induce preoperative radiological and clinical shrinkage of aneurysmal bone cysts [2, 47]. Continuous blood tests will, however, be necessary to monitor hyper- and hypocalcaemia [2].

### Limitations of this study

A general problem of systematic reviews and meta-analysis is the heterogeneity of the study quality included. Another weakness of the methodology of this publication could be the selection of papers on different treatment concepts and 2 different entities (JBC and ABC).

## Conclusion

Our meta-analysis showed that surgical intervention of both aneurysmal and juvenile cysts yielded good to very good results. For curettage it was apparent that type and quality of the interventions were of critical importance and that combining it with (autologous) cancellous bone graft reduced the number of recurrences.

Cyst resection for aneurysmal bone cysts led to very good outcomes whereby the term ‘resection’ is used rather loosely thus blurring the extent of the intervention.

Elastic intramedullary nailing seems to achieve excellent outcomes in the management of juvenile bone cysts but additional research studies would be required to permit a valid recommendation.

Injection methods in the treatment of aneurysmal and juvenile bone cysts must be clearly differentiated. Excellent outcomes were achieved with both Doxycycline injections and injection of alcohol into aneurysmal bone cysts. Our meta-analysis raised the question of publication bias in regard to Doxycycline injections; alcohol injection, however, was associated with numerous complications.

The method of steroid injections in the management of juvenile cysts was affected by the far-reaching heterogeneity of the individual studies. Furthermore, as for all injection procedures under review, several treatment cycles were required to accomplish a consolidation of the cysts. A valid recommendation for the other injection procedures cannot be formulated because of the low number of available studies and/or the small collective of cysts under investigation.

Radiotherapy alone in the treatment of aneurysmal bone cysts does not appear to be an advisable treatment option because of the high complication and recurrence rates.

Selective arterial embolization is inferior to surgical intervention based on its outcomes as well. However, the neoadjuvant application appears to reduce the number of recurrences in the treatment of aneurysmal bone cysts and prevent or minimize bleeding complications.

Non-surgical management of juvenile bone cysts led to very poor results but could be consider in individual cases and after pathological fracture. In fracture situation, the decompression of the cyst may possibly lead to better outcomes.
